# Caring During COVID-19: Reconfigurations of Gender and Family Relations During the Pandemic in Switzerland

**DOI:** 10.3389/fsoc.2021.737619

**Published:** 2021-11-05

**Authors:** Nolwenn Bühler, Mélody Pralong, Cloé Rawlinson, Semira Gonseth, Valérie D’Acremont, Murielle Bochud, Patrick Bodenmann

**Affiliations:** ^1^ Department of Vulnerabilites and Social Medicine, Unisanté, University Center for Primary Care and Public Health, Lausanne, Switzerland; ^2^ Institute of Social Sciences, University of Lausanne, Lausanne, Switzerland; ^3^ Unisanté, University Centre for Primary Care and Public Health, Lausanne, Switzerland; ^4^ Faculty of Biology and Medicine, University of Lausanne, Lausanne, Switzerland

**Keywords:** care activities, COVID-19 pandemic, gender inequality, moral responsibility, protective measures, safeguarding role, Switzerland

## Abstract

COVID-19 caused major changes in private and public arenas. Individuals were forced to reorganise their daily lives in response to the restrictive measures imposed by governments. The redistribution of gender roles and the responsibility for care provides an example of the reconfigurations that took place during the pandemic. This article sheds light on the implications of the pandemic for gender inequalities by exploring how care work was reconfigured as women and men sought to protect family members and navigated risks of infection. The study is based on qualitative data – interviews and observations – gathered in an interdisciplinary medical anthropology project. In the article, the authors focus on seven cases selected from a larger corpus to illustrate how reconfigurations of the gendered division of care work within families shifted during the pandemic as men assumed greater moral responsibility for safeguarding family members, without infringing the norms of masculinity. The first part of the article explores the intensification of care activities during lockdown for women living in the Canton de Vaud in Switzerland. The second part centres on the moral responsibility and duty for women and men to protect family members from viral exposure. The results from the study confirm not only that most care activities continued to be delegated to female family members, but also that men’s roles evolved. While their safeguarding role can be understood as a new form of caring for men, the findings suggest that it was essentially crisis specific and did not challenge masculinity norms. The extent to which this reconfiguration of gender roles might have a longer-term impact on gender inequalities remains to be seen. Meanwhile, these observations could have important implications for policies aimed at mitigating the medium and long-term effects of the pandemic on gender inequality.

## Introduction

The COVID-19 pandemic placed poorer countries and specific population groups at greater risk of suffering from the short and long-term consequences of the global public health crisis. It drew attention to persisting health and social inequities, especially for groups most affected by structural vulnerabilities (Bourgois et al., 2017). As analyses of previous epidemics, such as Ebola or Zika, have shown, women are more likely to be affected by the mitigation measures imposed by governments, especially if they are already facing structural vulnerabilities (Davies and Bennett, 2016; Menéndez et al., 2015). Studies based on statistics from the US, China and Europe (Ryan and Ayadi, 2020) reported immediate and potential longer-term impacts of the pandemic on girls and women, including gender-based violence, economic dependency, school drop-out rates, access to reproductive health services and job losses. As COVID-19 infections and death rates progressed, women, and especially those from ethnic minorities or precarious socio-economic backgrounds, were found to be more severely affected by mitigation measures than men. The differential impact on women was due to structural factors associated with the over-representation of women on the frontline in the provision of essential services, as workers in the formal and informal care economy (Smith et al., 2021), and as home carers, nurses or in retailing, leaving them at greater risk of exposure to the virus (Bahn et al., 2020). In the early stages of the pandemic, researchers in Switzerland, one of the wealthiest countries in the world, expressed concern about increasing inequalities, especially at the intersection between categories of gender, social class and ethnicity (NCS-TF, 2020).

Gender researchers, especially materialist feminists and political economists, have shown that the gendered division of labour in the domestic sphere is central in the production and perpetuation of intersecting inequalities, and they have shed light on the power dynamics at stake in its delegation and distribution (Delphy et al., 2019; Falquet et al., 2010; Hirata and Molinier, 2012). Care work is understood both as an activity involving taking care of/caring for other people and feeling concerned about others (Laugier, 2016). To take care of and to care about someone or something means paying attention to the ordinary needs of others through “unnoticed, invisible tasks” (Laugier, 2016, p. 211). Care work has long been mainly carried out by women and confined to the domestic and private spheres. The exclusion of these activities from the public arena has reduced them to “private sentiments devoid of public moral and political import” (Laugier, 2015, p. 219). Whereas care work is considered essential and fundamental for human life, it has been historically depreciated, materially and symbolically, and has remained invisible and undervalued (Sayer, 2005). The feminist ethics of care (Laugier, 2016) invites us to think of care activities in relation to vulnerability and interdependence, not as specific characteristics of some groups of the population, but as an ordinary, yet crucial, part of life.

Recent studies in France (Boring et al., 2020; Collectif d’Analyse des Familles en Confinement, 2021), Italy (Del Boca et al., 2020), and the US (Umamaheswar and Tan, 2020) highlight the increased importance of paid and unpaid care work for the functioning of societies and families. Public health restrictions imposed a significant re-organisation of life in the private sphere, especially regarding care activities. The impact of the pandemic on care work and gender inequalities became a much-debated issue. It opposed authors with a “pessimistic view”, who insisted on the increased burden of care work on women, and those who considered the pandemic as an opportunity to reshape gender relations and roles more equitably. While lockdown enabled a possible redistribution of activities and responsibilities related to care, findings in France show that women took on a greater responsibility for care activities and faced an increased workload in terms of housework and childcare (Collectif d’Analyse des Familles en Confinement, 2021), a finding also salient in Switzerland (Widmer et al., 2020).

In Switzerland, before the pandemic, women were primarily in charge of activities relating to the household: in 63% of households without children, and between 71.6 and 80.6% of households with children, depending on the age of the mother (Berrut et al., 2018). Women were shown to be the main providers of childcare in 80% of households with children under the age of 13. In the context of COVID-19, a study carried out in Switzerland compared the situation before the pandemic and after the first wave using data collected between September 2019 and March 2020, and then between May and June 2020 (Refle et al., 2020). The study indicated that parents with children under the age of 18 increased the time spent on household and care activities, with a slightly greater increase for mothers than for fathers. No change in the level of satisfaction was reported regarding how these activities were shared between partners before and after the outbreak of the pandemic: 8.4 score out of 10 for men and 7.7 for women. Women initially carried out more of these activities than their male counterparts. While the increase in time spent on care activities by fathers rose from 13 to 17 h per week during lockdown, it increased from 30 to 39 h for mothers.

Caregiving roles and activities also became more apparent, in relation not only to gender inequalities, but also to the perception of risk and the adoption of protective measures. Studies in diverse countries, such as Bangladesh (Ferdous et al., 2020), Spain (Vega et al., 2020), China (Zhong et al., 2020), Turkey (Yıldırım et al., 2021), and eight OECD countries (Austria, Australia, France, Germany, Italy, New Zealand, the United Kingdom and United States) (Galasso et al., 2020), reported that women were more inclined to adopt and adhere to protective practices. Women also tended to perceive a greater risk related to COVID-19 and applied protective measures more strictly (Galasso et al., 2020; Vega et al., 2020). Women seemed to be more concerned about their loved ones, and generally worried more about potential adverse effects of the virus, while men were more preoccupied with the effects on the economy and on society (Czymara et al., 2021; van der Vegt and Kleinberg, 2020). These differences do not always appear to be related to gender differences per se (Umamaheswar and Tan, 2020). Carrying out and being responsible for care activities affected attitudes towards risk and protection, and more importantly, generated anxiety and distress for those bearing them, whether they be men or women in charge of adopting caring roles (Umamaheswar and Tan, 2020). These studies suggest that attitudes towards risk and protection depend primarily on the engagement of the person bearing the responsibility and performing the “duty of care”.

As epidemiological studies have shown, the domestic space is a privileged site for transmission (Dupraz et al., 2020), putting extra pressure on family members to negotiate risk in their daily lives, especially when several generations share the same space. The literature in early 2020 did not show clearly how care activities, borne mainly by women, and more broadly gender and intergenerational dynamics, affected and intersected with viral exposure and protective measures. The objective in this article is to gain a greater understanding of the reconfigurations of care activities in the daily navigation of risk and protection, and to shed light on its implications for gender inequalities. The authors examine to what extent these reconfigurations reproduce “gender norms defining women as caregivers – nurturing, self-sacrificing, and caring – and men as breadwinners” (King et al., 2020, 80), as well as the implications for the management of risk and protection for both women and men.

The authors address two main research questions. In the first part of the article, they ask to what extent the pandemic and measures to contain it intensified care activities for women in the Swiss canton of Vaud during the first lockdown. The second part asks how the moral responsibility and duty to protect family members from viral exposure was distributed between men and women and led to the creation of a new role for men.

Analysis of the sharing of care activities shows how the pandemic provided an opportunity for men to take responsibility for safeguarding their families, representing a new form of “caring masculinity” (Wojnicka, 2021). The ambivalence of this role is explored here to determine whether it allowed men to adopt a caring role without infringing the norms of masculinity. The conclusion highlights possible implications for public health policies aimed at mitigating the medium and longer-term effects of the pandemic on gender inequalities.

## Methods

SociocoViD is a medical anthropology research project based on the exploration of daily life experiences of COVID-19 in the Canton of Vaud, Switzerland. The project was developed as part of an epidemiological project on transmission of, and immunity to, SARS-CoV-2 (SérocoViD). Canton of Vaud has densely populated areas and, with Geneva, was one of the areas hardest hit by the pandemic during the first and second waves (O’Sullivan et al., 2020; ATS, 2021). The Swiss health system is organised federally, and Cantons are in charge of health management for their population. Except for the state of emergency declared at the beginning of the pandemic, during which the Federal Council took over, the pandemic was managed at cantonal level. Measures implemented to contain its spread varied from one Canton to another. The Vaud Canton was chosen for the study both because it was one of the most affected epidemiologically, and because of its variety in living conditions: some people resided in densely populated zones and others in rural areas. The project was funded by the Swiss National Science Foundation (NRP 78 COVID-19, SNSF). The qualitative approach aimed to complement the epidemiological quantitative project by providing evidence about how living conditions affect viral exposure, the adoption of protective practices, and the emergence of additional vulnerabilities; and, in turn, how they affect health differentials and inequities across gender, ethnicity, and socio-economic status. The project combined syndemic (Singer, 2009) and intersectional approaches. Health differential categories were captured not as singular independent variables but as they intersect in complex and situated ways to exacerbate experiences of illness and health (Hankivsky and Christoffersen, 2008; Christensen and Jensen, 2012; Heard et al., 2019).

By focusing on individual experiences, the aim was to show how these structural inequalities materialised and affected people in their daily lives during the COVID-19 pandemic when they faced the risk of viral exposure and adopted protective practices. Data were collected using qualitative semi-directed interviews, complemented with ethnographic observations of participants’ living conditions. The intention was to carry out interviews in the participants’ homes but, due to the pandemic, participants were also offered the opportunity to be interviewed by video conference or in the building of the Centre of General Medicine and Public Health (Unisanté) in Lausanne. If participants chose not to be interviewed in their homes, they were asked to describe their living conditions in detail, and/or to draw them schematically, to enable the researchers to understand their material and spatial living arrangements.

Participants were selected from the registry of those who took part in the SérocoViD epidemiological study. Three groups of the population were included:

1) Index cases who tested positive for COVID-19 between 5 March and 2 April 2020, corresponding to weeks 2–5 after the first COVID-19 confirmed case in the Canton of Vaud, and members of the general population who were randomly selected;
2) Asylum seekers residing in an Etablissement Vaudois d’Accueil des Migrants (EVAM centres), which provides temporary accommodation for migrants in the Canton of Vaud;
3) Employees in essential non-medical services, including postal services and grocery stores.

The aim was to interview 15–20 participants in each group. To qualify for the study, participants had to be over the age of 14, registered as a SérocoViD participant, and able to speak French sufficiently well to take part in an interview, or agree to the presence of an interpreter.

Approval from the Ethics Committee of the Canton de Vaud was granted in May 2020 and then amended in December 2020 to include groups 2 and 3. When selecting participants in group 1, factors such as gender, age, number of people living in the household, type of housing and surface area of dwellings were considered to achieve a diversified sample of living conditions and experiences. For group 1, participants were contacted by phone to inquire about their interest in participating. For groups 2 and 3, contact was made with the responsible administrators of the EVAM centres and human resource departments of the selected enterprises, respectively. This approach facilitated access to populations who tend to be under-represented in large studies where participation is voluntary. For these two groups, interviews were mostly carried out directly on site.

At the time of writing (August 2021), 56 interviews had been conducted. Data collection was completed for group 1, N = 20, and for groups 2, N = 13, and 3, N = 22. On average, interviews lasted around 1 hour (between 40 min to nearly 2 hours depending on the participant). They were conducted by one or two members of the research team. Two of the interviews were conducted by video conference and one by telephone. All other interviews were conducted face-to-face. Of these, three were carried out in the institutions’ building. All other interviews were conducted at the participants’ homes, in the EVAM centre or the sector’s facilities. In cases where the participant did not have a sufficient knowledge of French, English or Spanish (especially for asylum seekers), translators were present during the interviews. Interviews were audio recorded and transcribed verbatim. Analysis was carried out using MAXQDA software. In cases where the interview was conducted in another language (Spanish or English instead of French), the researcher who carried out the interview translated the extracts included in this paper. In the article, names of participants have been replaced to respect their anonymity. The interviews covered several topics, but this article focuses on the data relating to gender and care.

## Results

The results presented here are based on the analysis of seven cases which explore reconfigurations of care activities and how they relate to the daily navigation of risk and protection, focusing on their implications in terms of gender. The cases were selected to allow more in-depth description and understanding of how the respondents reconfigured their lives by taking account of the practical and moral caring responsibilities they assumed during COVID-19, especially during the first lockdown. The first topic is represented by one case – Claire, a single mother aged 51 – to illustrate the intensification of care activities as a moral responsibility assigned to women, and confirming the increased burden of care for women, as already shown in numerous studies (Czymara et al., 2021; Fodor et al., 2021; Hupkau and Petrongolo, 2020; Jessen et al., 2021). The second topic explores the emergence of a new safeguarding role, often assumed by men to protect their families from viral exposure, based on six cases: Alberto, aged 31, living in an EVAM centre in the Canton of Vaud, with his pregnant wife, and their 3-year-old daughter; Eva, aged 36, also living in an EVAM centre with her disabled son; Sylvie, a retired woman aged 69, living with her husband in a house in a semi-urban area, and who has two adult sons, Filip and Ben, aged 32 and 34 married with children, working in “essential services” and living in apartments; and Robin, a young man aged 17 studying at a secondary school, living with his parents and his 12-year-old brother in a house in a semi-urban area.

### Intensification of Care Activities: A Moral Responsibility Assigned to Women

Switzerland opted for a “flexible lockdown”, which was in force between 16 March and 27 April 2020. The Swiss version of lockdown was “flexible” in that, unlike other neighbouring countries (Italy, France, Germany), the population was advised to stay home but was allowed to move freely within the national territory. This flexibility also characterised the management of the pandemic adopted by Swiss federal authorities, which relied on individual moral responsibility to navigate risk and protection in daily life in the name of collective solidarity. Pre-existing structural inequalities in living conditions, including gender, meant that not all individuals benefitted equally from this flexibility. During lockdown, the domestic and private spheres became the main living environment for many people. The closure of most services increased the amount of time and activities household members spent together in their homes. Parents could not rely on the distribution of care activities between formal and informal settings (schools, childcare institutions), or people living outside the household (grandparents, home helps, daily care workers). Families had to re-organise their everyday lives to manage family, professional and domestic life simultaneously in a unique shared space.

Claire’s experience sheds light on how the pandemic intensified daily care activities in both the professional and the private spheres as protective practices extended outside the household in multiple ways. Claire is a teacher in a primary school with two classes of pupils. She is also involved in care activities in her private life. Within the domestic sphere, she is a single mother who takes care of her three children, aged 15, 19 and 21, living at home. She also takes care of her parents on a regular basis. When schools for all age groups closed from March to May 2020, Claire found new ways of continuing to care for her young pupils (aged 3–5) in spite of the restrictions, when her school’s closure was announced on 13 March 2020, Claire remembers feeling her smartphone vibrate in her pocket and reading the news alert:

Oh! I could have just cried in front of my pupils, it was so … so intense, they were all around me and were telling me: “It’s OK teacher, we will see each other soon!” And I had to hold on and say: “Yeah it’s OK, we’ll see each other.” I found that we (teachers) were really not well prepared for that.

The unprecedented measures imposed by authorities revealed the seriousness of the crisis. Facing a very unusual situation, Claire and her pupils took care of each other by trying to reassure themselves and hoping for a reunion in the near future. Claire pursued this work of maintaining caring relationships even though she was physically away from her pupils, illustrating the relational and moral dimension of care activities. She had to adapt her way of teaching, be resourceful and brave, despite the uncertain situation that everyone was experiencing:

It was really complicated during the first two weeks (after the beginning of the first lockdown) and then I managed to handle my work. I created a website, and I gave the link to my pupils. It allowed me to upload stories, crafts ideas, links for the parents, stay connected.

Claire decided to provide her pupils with some pedagogical material, even though she was not required to since they were very young, and no formal assessments were expected for their ages. By creating a website, Claire assumed a moral responsibility to care for her pupils by maintaining a relationship with them as well as their parents during lockdown. She searched for ideas, activities, made and received phone or video calls from her pupils, created a WhatsApp group for all the parents, managed technical problems, and adapted the website to the needs and uses of the families. Some parents were grateful, enthusiastic and even uploaded their own material onto the website, whereas others did not have time to explore the platform. She observed that not all pupils were able to access the material, and some had better access than others. Creating a website seemed like the most manageable solution for Claire to reach as many pupils as possible and their families. Maintaining this connection was practical, relational and time-consuming work that needed to be organised in conjunction with the care she was also providing for her own children in the domestic sphere:

Simultaneously I had to ensure that everything was going OK at home, that nobody [her three children] worried too much. We found a rhythm, and that was fantastic. We walked a lot, I was thankful to our health authorities that they let us go outside … because here it was a bit difficult, we have a very small balcony. And with three children, there were often fights in the kitchen.

Because Claire and her three children spent more time together in their apartment, tensions surfaced more quickly than usual between them. Claire’s family needed to find a new daily “rhythm” to manage the increased relational difficulties that her family was facing in sharing the same living space. This situation was complicated by her daughter’s job in a hospital where she had just started an internship and was experiencing considerable anxiety and stress with only limited supervision at the workplace:

She had a job (outside the home), with all the fear that it caused, the difficulty of leaving in the morning and the fact that her supervisors themselves worked from home. So, she was there, and she had zero knowledge and experience and had to manage complicated situations with her supervisors working from home. That was difficult. So, we [Claire and her other children] had to make her feel better. We spent the evenings asking her: “Do you want to keep working there, do you feel it’s going to be OK, do you want us to ask [the hospital] if you can stop working?”

The risk of exposure or transmission associated with her daughter’s work was not Claire’s main concern; rather, she worried about the difficulty of balancing a stable relational equilibrium between all members of the household in a situation of anxiety, stress, and growing tension. For Claire, care for her children involved the moral responsibility of providing and ensuring a safe relational environment for them.

Besides her pupils and her children, Claire’s care activities also extended to vulnerable others outside the household. During the first lockdown, she offered her help to a female friend, who was struggling to manage both work and family life. She offered to take care of her friend’s father, who was also her neighbour, by bringing meals to his mailbox. In this situation, she took care both of a vulnerable neighbour, whose age required him to stay home, and of her friend, who was struggling to manage her care responsibility. Claire did the same for her own parents, on her own initiative. She brought groceries to them and left them in front of their door in the first weeks of lockdown, but she quickly stopped because they decided to go back to the supermarket, even though she insisted on doing their shopping to protect them. Claire reached out to others in new ways and helped them with their practical and affective needs, which sometimes meant reconfiguring relations and reinforcing emotional ties, as with her parents:

We said “I love you” more to each other. “Take care of yourselves.” Because if my parents would have died … they are old, they were quite ready. We talked about it. My dad says readily: “I’ve lived my life.” But the fact was that they were quite isolated, that they couldn’t see anybody, and they were still a little bit afraid … So we said: “It’s going to end soon.” Something to give us courage.

Claire’s experience illustrates in an exemplary way the temporal continuity of caring in family life as essential work mostly done by women. Claire’s work was primarily relational and affective, requiring creativity and strength of purpose. It entailed an intensification, in both quality and quantity, of her temporal and affective availability, shedding light on the unlimited and endless character of care activities, which she assumed alone as the main carer of the family. Her care work not only intensified temporally, practically, relationally and emotionally, but the number of others to care for also increased, and the sphere of people that she was caring for also expanded. Her experience reflects the ways in which women continued to assume caring responsibilities — unlimited in time and requiring unlimited energy — and normalised them. Claire’s care activities increased in the domestic, parental and professional spheres during the first lockdown but did not cease once the public health restrictions were lifted. A hypothesis to explain why care activities did not return to pre-pandemic levels is that women are dealing with the unintended emotional and relational effects of the pandemic which continue to be felt even when restrictions were partially eased.

### Protecting the Family from Viral Exposure: The Emergence of the Safeguarding Role

The selected interviews illustrate the emergence of a new configuration of the caring role which focuses on mitigating the risk of viral exposure and protecting the family from exposure to the virus, involving the duty to care for the family in the face of external threats generated by the pandemic. Extracts from the interviews show how this role was experienced and negotiated by six different women and men during the first wave of the pandemic.

Alberto and his family live in an EVAM centre, where, on most floors, residents live in separate rooms and share the kitchen and bathroom. Because his wife was pregnant and, therefore, more at risk of developing severe symptoms of the disease, Alberto tried to protect her, the baby to come, as well as their daughter, from viral exposure within the centre. He describes this responsibility as something highly unusual and as an important source of anxiety for him:

Our living space was very small, the hardest part was that the kitchen and the bathroom were outside our room, so we were facing an increased risk of being contaminated. I remember that, on the same floor as us, someone got COVID, and we became psychotic [laughs] for a while, when we had to shower and thus enter the shared bathroom or kitchen. I felt like a controller, I almost had the bottle of hand sanitiser attached to my belt, I remember that I was really attentive to other people’s gestures [laughs]. Sometimes I saw the person who had the virus going to the kitchen or the bathroom. So, if I knew that my wife was going to go there to cook or something else, I used to get there first and I cleaned everything, I put disinfectant on everything.

The pandemic turned ordinary living spaces into unsafe environments, and this risk increased with the number of people who shared and used the same space, suddenly generating new forms of emotional and physical vulnerability to viral exposure. To ensure that his family could continue to use these spaces and remain protected from viral transmission, Alberto assumed a safeguarding role comprising the task of “cleaner”, but also of “controller”, as he described it. Shielding his family’s health required taking care of material spaces, and paying attention to others, who became potential threats to his family’s well-being. Alberto’s care activities aimed to mitigate the potential risk of exposure and infection in daily living spaces by maintaining safe boundaries around his family members. By caring for places through ordinary tasks such as cleaning and sanitising, Alberto protected his family from being contaminated. He also adopted a surveillance role, paying attention to those who respected protective practices and those who did not, enabling him to keep track of potential risks of transmission. He acted primarily to protect his pregnant wife and child by maintaining a *cordon sanitaire* around them.

This safeguarding role was also assumed by Eva. She explained how she started to pay more attention to her physical environment and to respect protective measures to maintain a safe space around her disabled son:

I go to the kitchen, I open all the windows, I leave the door open. We don’t have a choice [to use the common spaces and avoid being exposed to risk] regarding what to do to protect ourselves. When my son takes a shower, I clean, of course, all the doorknobs, the shower and all, but we cannot protect ourselves completely. First, I didn’t want my son to play [with other children at the centre] because the children and the people here, they were outside all the time, they had visitors in their rooms, even my neighbours, sometimes five or six people in their room, next to ours. So, at first, I didn’t want my son to play with them. That’s why it was always just the two of us together, because I wanted to protect my son.

Analysis of this new safeguarding role sheds light on the dual facet of care, as “controlling” and “protecting”. By paying greater attention to the environment, both Eva and Alberto sought to maintain a safe boundary around vulnerable family members to prevent them from viral contamination.

The pandemic showed that caring for others sometimes means staying away from people. In Alberto’s and Eva’s cases, it implied cultivating a relational and physical distance from the other residents in the centre, but it could also mean staying away from members of their families. Sylvie experienced such a reconfiguration of her family relationships during the first wave of the pandemic. Both she and her husband are retired. Sylvie has close relationships with her children (both adults) and grandchildren and feels supported by them and cared for. She remembers how her sons assumed a safeguarding role in trying to protect her at the beginning of the first wave of the pandemic:

At the beginning, I must say that one of my sons warned me, he told me: “Listen mom”, I think he was going on the internet, “it’s serious, it’s serious what’s going to happen, you need to make provisions”. I told him: “Come on, really?” He told me: “Yes, yes, listen to me.” What really made me realise [that the situation was serious] was the behaviour of my children because my other son told me: “You are not going to the stores anymore.” And besides I think we [persons over 70] were told to stay at home, and they [her children] brought us food and told us to leave them for 1 hour in front of the door before taking them inside. It didn’t make me more anxious, but I thought: “OK, it is really really serious.”

In contrast to Alberto’s and Eva’s experiences, Sylvie’s safeguarding role served outside her home to implement protective practices for herself and her husband. Safeguarding their parents’ health required Sylvie’s sons, first to convince her and her husband of the seriousness of the risk associated with viral transmission, and then, practically and materially, to organise their daily life to maintain the *cordon sanitaire* around their living environment and avoid possible exposure to the virus. Finally, the work of protection was not limited to managing the risk of viral transmission by taking on grocery shopping and the maintenance of a safe material environment for their parents; it also extended to filtering other forms of emotional exposure. As all TV channels where focused on COVID-related news, this could generate feelings of anxiety and a sense of being overwhelmed. Sylvie’s sons discouraged their parents from watching TV news and insisted on the potential threat it represented for their mental health, thereby extending the safeguarding role to all “toxic invasion” in the viral or emotional form. In this case, the pandemic reconfigured the intergenerational relationship in a way that reversed the ascendant flow of care, as their sons took care of their parents whereas, before the pandemic, Sylvie felt that she took care of them and of her grandchildren.

Here too, the safeguarding role is ambivalent. Her son adopted behaviours usually assigned to women for the sake of his parent’s protection, at the same time as deciding what is good for them and what is not and controlling the boundaries. The way Sylvie describes the reversal of the hierarchical relationship between parents and children demonstrates how she came to accept the protective roles assumed by her sons which was justified by her “vulnerability”.

The risk of viral transmission to family members was a particular concern for individuals who had to continue to go to their workplace during lockdown. Filipe and Ben who both worked in food retailing stores provide such an example. Filip saw himself as a potential threat for his vulnerable family — his wife, pregnant at the time, and his young daughter — and adopted strict rules and hygienic measures to protect them:

I had no choice, I had to earn money. Regarding protective measures, when I got home [after work], I didn’t go near them [his wife and child]. I used to go to the bathroom, take off my clothes. I had my pyjamas ready, or shorts, and I left the clothes [worn during the day] in a room where no one used to go. We had to put rules in place, you never know, to protect my wife. If I contract it [the virus], I wouldn’t worry, but it was for her, she had a baby, and even for my daughter.

Similarly, Ben’s experience sheds light on the adoption of the same protective practices. As soon as Ben got home from work, where he was in contact with a large number of people, he did not kiss his wife and daughter. He took a shower and changed his clothes before hugging his family. This “decontamination” process, as he described it, enabled him to protect his family but created a difficult relational and emotional situation, where he had to stop his daughter from hugging him:

It’s difficult and weird because the first thing you want to do (when you get home) is hug your daughter. I had to do it … and we know it’s temporary.

Robin, a young man aged 17 studying at a secondary school was living with his parents and his younger brother aged 12 in a house in a semi-urban area. He described his living arrangement as privileged and very comfortable, due to the large rooms and the garden surrounding the house. When asked about how he experienced lockdown, he described the work his mother did to build and maintain a new family organisation, including the preparation of regular lunches, which was rare for a family whose members were used to eating at school or work at midday. Robin also mentioned his father’s health status. Because his father had a heart condition and was more at risk of developing severe symptoms if he contracted the virus, he imposed very strict rules on his family. Robin and his family complied by self-isolating during the first wave of the pandemic. Robin understood the importance of reducing social contacts and adopted protective practices to ensure the house provided a secure environment. He restricted the amount of socialising by limiting the number of contacts with his friends, but he also re-oriented it towards outdoors spaces. He spent a lot of time practising sports outside with his friends and fully adopted and adhered to social protective measures. In this family configuration, the safeguarding role was applied by the father in a strict and authoritarian way. But, as a result, Robin also became involved in safeguarding practices because the role was imposed on him by his father’s condition.

When the restrictive measures started to be lifted, Robin and his father negotiated new forms of living arrangements that would maintain a sense of security: Robin began seeing his friends again but kept a physical distance from them. His experience of this unusual, yet necessary, arrangement was positive:

So he allowed me to see my friends again, but I kept the distance, and I was really OK with that, because I could see my friends, we were outside, the weather was nice. I was the one [among his friends] who had to be the most careful because there weren’t people at risk in my friends’ families, so they didn’t keep a distance between them, but they did with me, and we know each other very well, we know each other’s parents. There wasn’t any trouble with that. We did not have the same reasons to act [regarding the protective measures]. The mother of one of my friends was away in the mountains [living in the family’s cabin] so he was living alone at home and didn’t need to keep the distance. But everybody was understanding.

Robin’s case illustrates the internalisation of the safeguarding role as an individual responsibility through which to navigate daily life and negotiate lockdown measures, according to individual social networks and living conditions such as access to outside spaces.

## Discussion and Conclusion

This article uses in-depth interviews to understand experiences and reconfigurations of care work during the COVID-19 pandemic in the Canton of Vaud, Switzerland where a flexible form of lockdown relied on the internalisation and individualisation of the responsibility to protect members of the household and the community. This approach is embedded in a moral economy (Fassin, 2009), in which people are expected to take responsibility for their own behaviour in the name of solidarity with groups of the population considered to be vulnerable. This form of lockdown trusts people’s common sense and ability to take the right decisions for themselves and for others, leaving room to adjust and navigate measures depending on their living arrangements and social relationships. This internalisation and individualisation of moral responsibility for navigating risk and protection can exacerbate inequalities between and within families.

By focusing on the reconfigurations of care work during the pandemic, the article shows how they are gendered in subtle and sometimes ambivalent ways, and how they affect the daily navigation of risks and protective actions. Care has been historically and socially assigned to women. It involves invisible, repetitive, daily work that requires constant availability, entailing a mental load (*charge mentale*) (Haicault, 2020) borne essentially by women (Collectif d’Analyse des Familles en Confinement, 2021). When men engage in care activities in the domestic sphere, they usually do so opportunistically in response to a visible task, and their availability remains conditional and subservient to other priorities. This unequal distribution of care work relates to specific forms of hegemonic masculinity (Mellström, 2020), ascribed to the role of the breadwinner.

The first part of the analysis in this article showed how the pandemic increased the moral, emotional, relational, and practical work of care usually performed by women. This work was intensified materially by the blurring of the boundaries between family and professional spheres, and emotionally by the number of others made vulnerable by the pandemic, in both cases requiring more time and energy on the part of women. Claire’s case confirms the observation that the increase in domestic and parental tasks has generally been absorbed by both parents, without changing the previous distribution of roles (Collectif d’Analyse des Familles en Confinement, 2021, p. 122).

The second part of the analysis illustrated the emergence of a new kind of work and safeguarding role to protect the family from viral exposure by building a *cordon sanitaire* around them. In the qualitative data, this role is performed in a diversity of living arrangements, age, gender, profession and socio-economic statuses. In the first case analysed here, the care activities described focus on the maintenance and continuity of affective relationships and daily life activities. Maintaining health and well-being of family members and her pupils at school was central to Claire’s conception of care work, rather than viral exposure per se. The risk of exposure to, and infection by, COVID-19, was not the main driver of her care activities, less because she was not concerned about exposure to the virus, but more because she was already fully occupied with the invisible and unlimited task of taking care of others and maintaining their well-being in their everyday lives.

By contrast, in the other six cases, the risk of viral exposure and the measures implemented to contain it took centre stage. The safeguarding role encompassed a set of activities that are usually defined as care and attributed to women, but it also involved other activities, which relate more closely to masculine norms, especially the expectation that men ensure the financial and physical protection of their families, illustrating the dual facet of care. On the one hand, safeguarding covers a range of domestic activities — cleaning surfaces, adopting strict rules of hygiene, grocery shopping for others — usually assigned to women. On the other, it consists of protecting family members from the risk of infection and transmission by implementing protective measures in more controlling ways. The safeguarding role has been interpreted as a form of “caring masculinity” (Wojnicka, 2021), which requires men to adopt care values, such as interdependence and relationality. However, it also aligns with more traditional norms of masculinity, relating to the social expectation that men are in control, but are not vulnerable themselves, and have the moral duty to provide security by shielding their families.

This new form of care is highly visible and crisis specific. The moral responsibility to protect one’s own family emerged temporarily as an everyday life “heroic” role for a man protecting his family from viral threats. Contrasting understandings of risk emerged. For women engaged in the intensification of care work, risk was understood in very broad terms as covering all the emotional, relational, physical and social elements contributing to well-being. For men, risk tended to be limited to viral contamination.

The findings complement qualitatively the quantitative results of the SérocoViD project by shedding light on the reconfigurations of care and gender in the daily navigation of risk and protection, which was not captured by the quantitative approach. They confirm the epidemiological importance of the household (Dupraz et al., 2020) as a key determinant in understanding how families managed viral transmission and protection. But what are the consequences of these observations for the management of the pandemic at national level? The analysis shows that women saw their practical and moral load increase during the pandemic. The emergence of a safeguarding role for men allowed them to combine male social normative expectations about managing, being in control, and protecting others, while at the same time enacting a form of caring masculinity. It may be crucial in pandemic times that a member of the household actively assumes responsibility for safeguarding family members. However, since this activity is visible, compared to the invisible continuous care work which continues to be performed by women, and borders on emotional and relational well-being, it might reinforce gender inequalities. The reconfigurations observed raise awareness of the need to enhance the value of care and to distribute it more equally, while also improving understanding of gender differences in care activities with regard to their temporal dimension, living arrangements, and social visibility.

If transferable to other contexts in Switzerland and replicated more widely, these findings could have important policy implications, especially when addressing the risk created by the pandemic of increasing gender inequalities. During a period when the essential importance of care for family well-being was so starkly revealed, attention has been drawn to the urgent need for concrete measures to address the moral responsibility for care and the burden it entails. At a practical level, the findings from this study confirm the need for women and other providers of care services to receive appropriate material and emotional support designed to mitigate gender inequalities. The extent to which the safeguarding role will be perpetuated in the longer term as the viral threat diminishes remains to be explored.

## Data Availability

The datasets generated for this study are held in the secured shared folder of the Unisanté internal server with restricted access to authorised SociocoViD and SérocoViD personnel. They are not available for external use for reasons of data protection and anonymity.
